# The R.E.N.A.L score’s relevance in determining perioperative and oncological outcomes: a Middle-Eastern tertiary care center experience

**DOI:** 10.1080/2090598X.2022.2064041

**Published:** 2022-04-17

**Authors:** Nassib Abou Heidar, Nizar Hakam, Jose M El-Asmar, Jad Najdi, Mark A. Khauli, Jad Degheili, Albert El-Hajj, Rami Nasr, Wassim Wazzan, Muhammad Bulbul, Deborah Mukherji, Raja Khauli

**Affiliations:** a Division of Urology and Renal Transplantation, American University of Beirut Medical Center, Beirut, Lebanon; b The Breyer Lab, University of California San Francisco, San Francisco, California, United States; c Massachussets General Hospital, Boston Massachussets

**Keywords:** Kidney cancer, renal cell cancer, RENAL score, kidney tumor, outcomes

## Abstract

**Objective:**

The aim of this study is to evaluate the significance of the R.E.N.A.L nephrometry scoring system in predicting perioperative and oncological outcomes and determining the surgical approach of choice for kidney tumors.

Patients and Methods: Our study retrospectively reviewed outcomes from the year 2002 to 2017. Mann-Whitney U test was used to compare continuous variables and chi-square test was used to compare categorical variables. Kaplan-Meier estimates and multivariable cox proportional hazard regression were performed to determine an association between the different R.E.N.A.L categories and disease recurrence or mortality.

**Results:**

A total of 325 patients underwent kidney surgery The most common R.E.N.A.L score category in our cohort study was intermediate (41.2%), followed by low, (33.2%) and high (25.5%). Patients with a high R.E.N.A.L score had worse perioperative outcomes compared to those with a low R.E.N.A.L score. High R.E.N.A.L score patients were 3 times more likely to receive blood transfusions compared to those with a low R.E.N.A.L score (19.4% vs 6.3%, p = 0.018), and a statistically significant longer hospital length of stay was also observed between the two groups (median 4.5 vs 4 days, p = 0.0419). In addition, the only predictor of disease recurrence or mortality was a high R.E.N.A.L score (Hazard Ratio (HR) 3.65, 95% Confidence Interval (CI) 1.05–12.7, p = 0.041).

**Conclusion:**

Our study sheds light on the use of R.E.N.A.L nephrometry score in predicting perioperative, postoperative, and oncological outcomes. Such findings may play a role in optimizing surgical approaches and pre-operative patient counseling.

## Introduction

Partial nephrectomy (PN) is the most widely utilized surgical approach in the management of stage 1 localized kidney tumors [1,2]. PN, whenever feasible, provides similar oncologic outcomes to radical nephrectomy (RN) while at the same time allowing for the preservation of functional renal parenchyma [3]. Widespread use of cross-sectional imaging has increased the overall incidence of renal tumors and has resulted in overall stage migration towards more localized kidney tumors [4]. The increased incidence of incidentally found small kidney tumors, in addition to the introduction of minimally-invasive surgical techniques popularized PN. However, Figure 2 the feasibility of PN is dependent on many patient-related variables as well as on certain tumor characteristics [5].

The decision to perform PN relies heavily on tumor characteristics; however, patients’ comorbidities and overall general health should also be taken into consideration during preoperative assessment. Older age, male gender, medical comorbidities (coronary artery disease, congestive heart failure, diabetes, and hypertension), smoking and obesity have all been shown to be associated with postoperative complications and decreased survivorship after PN [6].

To objectively evaluate tumor complexity, the R.E.N.A.L nephrometry score has been introduced as a standardized tool that relies solely on anatomical variables. These variables include mass radius, percent volume of the mass that is exophytic, proximity to renal sinus, and location [7]. Other nephrometry scores have been devised as well including the C-index, which estimates the mass’ proximity to the renal center [8], and the PADUA score, which utilizes similar characteristics as the R.E.N.A.L scoring system such as tumor size and location [9]. Multiple studies have shown close association between the aforementioned tumor complexity scores and operative difficulty, peri-operative complications, and intraoperative conversion to RN [10,11]. Their use in preoperative assessment has been vital in the decision making towards undergoing partial versus RN.

The aim of this study is to assess the impact of the complexity of renal tumors, stratified by the R.E.N.A.L nephrometry scoring system, on surgical outcomes, postoperative complications, as well as oncological outcomes in patients undergoing PN versus RN at a single institution, coinciding with adoption of robotic assistance.

## Methods

After institutional review board approval, a retrospective chart review was performed for patients with renal cell carcinoma (RCC) who underwent surgical treatment at a tertiary care center. De-identified data was collected for patients treated between 2002 and 2017 to allow a minimum of 3 years of patient follow-up. Data extracted included clinical parameters such as age, sex, medical comorbidities, tumor pathologic characteristics (tumor size, stage, Fuhrman grade, histology, margin status), surgical characteristics (type of surgery (partial vs radical nephrectomy), approach (open vs laparoscopic vs robotic), estimated blood loss, warm ischemia time), pre-operative renal function, and peri-operative complications. R.E.N.A.L nephrometry score was calculated for all patients and categorized into 3 groups: low (≤6), intermediate (7–9), and high (≤10).

Follow- up visits consisted of one post-operative visit at 1–2 weeks and following that, two visits per year for follow up cross sectional imaging. Disease recurrence was defined as any local or distant tumor detection with or without histopathological confirmation.

Descriptive statistics were reported as frequencies and percentages or medians and interquartile ranges (IQR). Univariate analysis was performed to explore associations between R.E.N.A.L categories and patient or tumor characteristics. Mann-Whitney U test was used to compare continuous variables and chi-square test was used to compare categorical variables. The yearly proportion of patients undergoing partial nephrectomy within each R.E.N.A.L category was determined, and trends over time were calculated using linear regression. Multivariable logistic regression was used to explore whether R.E.N.A.L score independently predicted surgery type thus influencing clinical decision making, with a priori adjustment for age, clinical T stage, pre-operative creatinine, medical comorbidities (coronary artery disease, hypertension, and diabetes mellitus). Model fit was assessed using Hosmer-Lemeshow goodness of fit test. Estimates of the probability of disease recurrence or mortality were calculated using Kaplan-Meier estimates. The log rank test was used to compare outcomes of patients with low, intermediate, and high R.E.N.A.L categories. Multivariable Cox proportional hazard regression was performed to test whether R.E.N.A.L categories were associated with disease recurrence or mortality, controlling for age, gender, pathological stage, grade, histology, and type of surgery. Statistical analysis was performed using Stata® version 16.1, and statistical significance was deemed at p < 0.05.

## Results

We identified 235 patients who underwent kidney surgery with complete data for analysis. The median age was 59 years (IQR 48–66) and 167 (71%) were males. None of the patients had a postoperative urinary leak. The most common R.E.N.A.L score category was intermediate (41.2%), followed by low, (33.2%) and high (25.5%). Table 1 depicts the clinical characteristics of patients, stratified by R.E.N.A.L category. Among patients with clinical stage T1a-2b, R.E.N.A.L category was associated with pathologic upstaging to T3 (1.4% in low vs 11.5% in intermediate and 9.8% in high, p = 0.041). In those upstaged to pT3, median R.E.N.A.L was 9 (IQR 7–10) vs 7 (IQR 6–9) in those without upstaging, p = 0.0408. Table 2 shows the post-operative outcomes and complications stratified by R.E.N.A.L categories. Patients with a high R.E.N.A.L score were 3 times more likely to receive blood transfusion compared to those with low R.E.N.A.L score (19.4% vs 6.3%, p = 0.018), and had a slightly longer hospital length of stay (median 4.5 vs 4 days, p = 0.0419).

**Table 1.** Clinical Characteristics of patients treated with surgery for RCC.

	R.E.N.A.L Category				
	Low	Intermediate	p	High	p
	(≤6)	(7–9)		(≥10)	
N	78	97		60	
Median Age (IQR)	58 (49–64)	60 (50–67)	0.2778	59 (47–69)	0.3879
Gender			0.563		0.258
Male	53 (67.1)	69 (71.1)		47 (75.8)	
Tumor size	3.2 (2.5–5)	4.5 (3.4–7)	**0.0019**	8 (6–11)	**< 0.0001**
Pathologic T stage			**0.002**		**< 0.001**
T1a	52 (67.5)	38 (41.3)		8 (13.8)	
T1b	13 (16.9)	31 (33.7)		16 (27.6)	
T2a	5 (6.49)	7 (7.61)		9 (15.5)	
T2b	3 (3.9)	1 (1.09)		5 (8.62)	
T3a	2 (2.6)	12 (13.0)		12 (20.7)	
T3b	0	0		3 (5.17)	
T3c	0	0		1 (1.72)	
T4	2 (2.6)	3 (3.26)		4 (6.9)	
*Missing*	*2*	*5*		*4*	
Fuhrman Grade			0.461		**0.032**
1–2	43 (55.1)	51 (52.6)		26 (43.3)	
3–4	19 (24.4)	31 (31.9)		27 (45)	
Unknown	16 (20.5)	15 (15.5)		7 (11.7)	
Histology			0.618		0.783
Clear cell	49 (63.6)	57 (58.8)		38 (61.3)	
Papillary	9 (11.7)	11 (11.34)		7 (11.3)	
Chromophobe	11 (14.3)	21 (21.7)		10 (16.1)	
Other	8 (10.4)	8 (8.2)		7 (11.3)	
Sarcomatoid	3 (3.8)	2 (2.06)	0.658	4 (6.45)	0.699
LVI	2 (2.53)	5 (5.15)	0.461	11 (17.7)	**0.002**
Surgery type			**0.004**		**< 0.001**
PN	59 (75.6)	53 (54.6)		7 (11.7)	
RN	19 (24.4)	44 (45.4)		53 (88.3)	
Median EBL (IQR)	200 (100–300)	200 (150–400)	0.1443	200 (100–300)	0.109
Transfusion	5 (6.33)	15 (15.5)	0.058	12 (19.4)	**0.018**
Positive margin	4 (5.06)	5 (5.15)	0.99	1 (1.61)	0.385
Ischemia Time	20 (15 − 25)	20.5 (12–27)	0.92	22 (17–25)	0.79

IQR: Inter-Quartile Range

EBL: Estimated Blood loss

**Table 2.** Peri-operative and post-operative outcomes stratified according to R.E.N.A.L category.

	R.E.N.A.L Category				
	Low	Intermediate	p	High	p
Median Ischemia time (IQR)	20 (15–25)	20.5 (12–27)	0.923	22 (17–25)	0.7929
Median EBL (IQR)	200 (100–300)	200 (150–400)	0.1443	200 (100–300)	0.109
Transfusion	5 (6.33)	15 (15.5)	0.058	12 (19.4)	**0.018**
LOS	4 (3–5)	4 (3–5)	0.7635	4.5 (3–5)	**0.0419**
Any complication	10 (12.66)	13 (13.4)	0.884	10 (16.1)	0.558
Readmission	4 (5.06)	3 (3.09)	0.702	0	0.131

IQR: Inter-Quartile Range

EBL: Estimated Blood loss

LOS: Length of Stay

**Figure 1.**
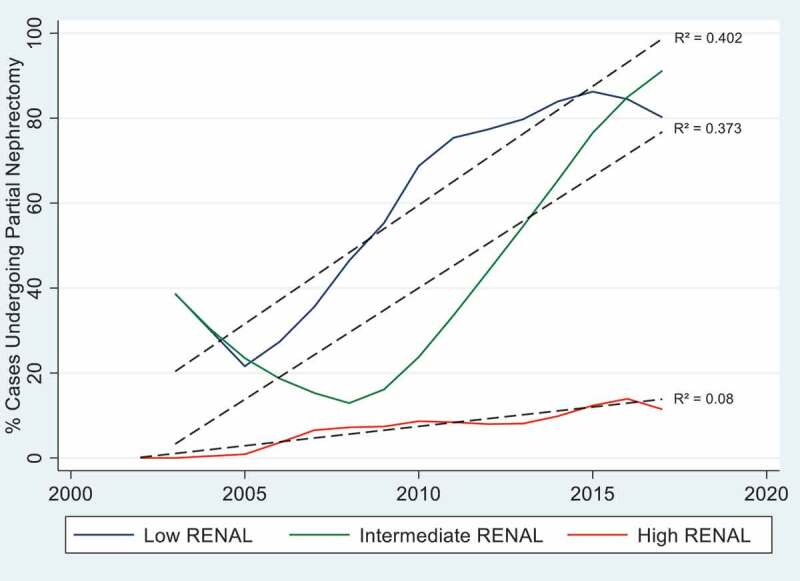
Percentage of Cases Undergoing Partial Nephrectomy Stratified by R.E.N.A.L Category.

The R.E.N.A.L score was also associated with choice of surgical procedure, where PN was performed in 75.6% of cases with low score, vs 54.6% of cases with intermediate score (p = 0.004), and only 11.7% of those with high score (p < 0.001). On multivariate analysis, after adjusting for age, clinical T stage, preoperative creatinine, and comorbidities, increasing R.E.N.A.L score by one category was associated with a stepwise decrease in odds of undergoing PN compared to patients with low R.E.N.A.L score (intermediate: OR = 0.41, CI 0.17–0.97, p = 0.042; high: OR = 0.05, CI 0.02–0.18, p < 0.001). However, Figure 1 demonstrates a significant trend over time of increased use of PN for patients with low category (p = 0.015, R^2^ = 0.402) and intermediate category (p = 0.016, R^2^ = 0.373), but not for high category (p = 0.294, R^2^ = 0.08).

**Figure 2.**
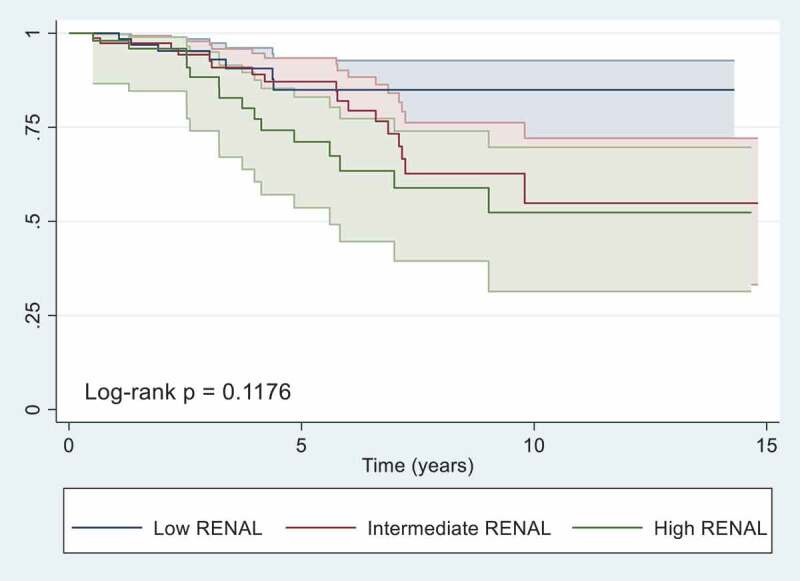
Kaplan-Meier Survival Curves with 95% Confidence Intervals Stratified by R.E.N.A.L Category.

Over a median follow-up of 4.4 years (IQR 2.3–6.9), 48 patients (20.4%) experienced disease recurrence or mortality. The overall 3, 5, and 10-year freedom of disease recurrence or mortality were 92% (CI 87% – 95%), 81% (CI 75% – 86%), and 62% (CI 51% – 72%). When stratified by R.E.N.A.L category, freedom from disease recurrence or mortality did not differ significantly (log-rank p = 0.1176). On multivariable analysis (Table 3), the only predictor of disease recurrence or mortality was high R.E.N.A.L score (HR 3.65, CI 1.05–12.7, p = 0.041), while an intermediate R.E.N.A.L score showed a statistically insignificant increased risk (HR = 2.34, CI 0.81–6.81, p = 0.117).

**Table 3.** Cox Regression Analysis Depicting the Predictors of Disease Recurrence or Mortality.

	HR	95% CI	p
R.E.N.A.L category			
Low	reference		
Intermediate	2.35	0.81–6.81	0.117
High	3.65	1.05–12.7	0.041
Age	1.01	0.97–1.04	0.685
Gender			
Female	reference		
Male	0.95	0.39–2.27	0.912
Surgery type			
RN	reference		
PN	2.12	0.72–6.25	0.174
p T stage			
T1a	reference		
T1b	0.22	0.04–1.14	0.071
T2a	1.54	0.45–5.21	0.487
T2b	1.07	0.11–10.4	0.956
T3a	1.64	0.48–5.67	0.431
T3b	1.46	0.18–11.8	0.72
T4	1.81	0.39–8.48	0.45
Grade			
G 1–2	reference		
G 3–4	1.99	0.89–4.48	0.094
Histology			
Clear cell	reference		
Papillary	0.18	0.02–1.45	0.108
Chromophobe	0.67	0.26–1.69	0.394
Other	0.59	0.12–3.01	0.53

## Discussion

In managing renal masses, the choice of procedure (PN or RN) and approach (open or minimally invasive) classically depends on qualitative data such as tumor anatomy, patient characteristics, and surgical expertise. Nephrometry scoring systems played a major role in quantifying and stratifying the complexity of renal masses. The R.E.N.A.L nephrometry score utilizes imaging related factors of renal masses to objectively assess tumor complexity aiding in the decision-making process of the elected procedure and type of approach used [7]. In patients undergoing open or minimally-invasive PN, the R.E.N.A.L score has been shown to be associated with perioperative outcomes [12,13]. Our study validates the utility of the R.E.N.A.L nephrometry score in predicting operative complexity and postoperative morbidity.

In keeping with available literature, our series shows that most patients with high R.E.N.A.L score tumors underwent RN as most of these tumors are anatomically complex and thus not amenable to PN [14]. Our data additionally reveals that when dealing with low and intermediate R.E.N.A.L score tumors there was an increased trend with time favoring PN especially since our institutions’ adoption of robotic surgery in the early 2010s. Similarly, Ali et al. report on a major increase in the use of PN for treating renal masses, up to 60% in 2016 from a previous 5% rate in 2010. Authors conclude that the increased use of the robotic surgery platform is the main culpable for the change in numbers [15]. It is quite evident that the introduction of minimally invasive surgery, namely robotic surgery, created a worldwide trend favoring nephron-sparring surgery which led to improved peri-operative outcomes even for highly complex tumors when compared to the traditional open partial nephrectomy technique [16].

In our series, patients with a higher R.E.N.A.L score tumors were more likely to receive blood transfusions and have an extended length of hospital stay. Similarly, a systematic review and metanalysis of 20 studies showed a clear increase in the incidence of postoperative complications including hemorrhagic ones and urine leaks for patients with higher R.E.N.A.L score tumors [17]. In addition, a higher R.E.N.A.L score was associated with a longer operative time which was in turn directly related to an extended length of hospital stay [18].

The impact of an RCC’s tumor complexity on oncologic outcomes is yet to be established. This study has shown that R.E.N.A.L score has a prognostic effect on significant oncologic outcomes. For instance, patients with low R.E.N.A.L score have a significantly improved survival rate when compared to patients with higher nephrometry scores. Similarly, a higher rate of recurrence and upstaging was revealed with high R.E.N.A.L score tumors. Previously, Kopp et al. showed that high complexity tumors (R.E.N.A.L ≥ 10) had a negative impact on progression-free survival [19]. Whereas, Weight et al. revealed that an increasing R.E.N.A.L score is associated with increased risk of mortality from RCC in addition to all-cause mortality [20]. Interestingly however, in another study by Hwanik et al., tumor radius was the sole factor associated with increased mortality [21].

Our study is not without limitations. To start, our sample consisted of a pool of patients before and after the adoption of robotic assisted surgery at our institution. Complications during the early adoption phase might be in fact related to surgeon expertise which could not be accounted for in our analysis. Moreover, we included a cohort of patients over a long period of time during which indications for PN changed dramatically as this technique was being refined and mastered. Moreover, radiologic data in nephrometry scoring was calculated manually by the authors, which is subject to human error. More importantly, the relatively small sample size of this study might not allow consequential and generalizable conclusions. Another limitation is that our cox regression model was likely underpowered to assess disease recurrence and mortality separately due to limited outcome events

### Conclusion

Our study reiterates the role of the R.E.N.A.L nephrometry score in predicting intra and postoperative complications highlighting its importance in pre-operative surgical planning. Additionally, our results shed light on the significance of a higher R.E.N.A.L score in predicting oncologic outcomes including recurrence and mortality. Future prospective studies investigating the correlation between R.E.N.A.L nephrometry score and oncologic outcomes are needed to further validate its prognostic role in RCC.
